# Bariatric Surgery Outcomes in Patients with Severe Obesity Compared to Patients with Non-Severe Obesity at A New Institution in The United Arab Emirates

**DOI:** 10.3390/jcm13071907

**Published:** 2024-03-26

**Authors:** Jamie P. DeCicco, Juan S. Barajas-Gamboa, Jerry T. Dang, Gabriel Diaz Del Gobbo, Javed Raza, Carlos Abril, Alfredo D. Guerron, Juan Pablo Pantoja, Safa Botros Hegazin, Ricard Corcelles, John Rodriguez, Matthew Kroh

**Affiliations:** 1 Cleveland Clinic Lerner College of Medicine, Case Western Reserve University, Cleveland, OH 44106, USA; deciccj@ccf.org (J.P.D.); abrilc@clevelandclinicabudhabi.ae (C.A.); corcelr@ccf.org (R.C.); rodrigj2@clevelandclinicabudhabi.ae (J.R.); 2 Department of General Surgery, Cleveland Clinic Abu Dhabi, Abu Dhabi 112412, United Arab Emirates; barajaj@clevelandclinicabudhabi.ae (J.S.B.-G.); gabodelgobbo@hotmail.com (G.D.D.G.); razaj@clevelandclinicabudhabi.ae (J.R.); guerrod@clevelandclinicabudhabi.ae (A.D.G.); pantojj@clevelandclinicabudhabi.ae (J.P.P.); hegazis@clevelandclinicabudhabi.ae (S.B.H.); 3 Department of General Surgery, Cleveland Clinic, Cleveland, OH 44195, USA; dangj3@ccf.org

**Keywords:** super obesity, non-super obesity, bariatric surgery, weight loss, sleeve gastrectomy, Roux-en-Y gastric bypass

## Abstract

**Background:** Bariatric surgery is an effective treatment for weight loss, but a higher body mass index (BMI) may lead to higher postoperative complication rates. This study aims to compare perioperative and postoperative outcomes between UAE patients with severe obesity (SO) [BMI ≥ 50 kg/m^2^] and non-severe obesity (NSO) [BMI < 50 kg/m^2^] undergoing primary bariatric surgery. **Methods**: From September 2015 to July 2019, 542 patients, 94 SO (56.5 ± 6.2 kg/m^2^) and 448 NSO (41.8 ± 4.1 kg/m^2^), were retrospectively reviewed. **Results**: Patients with SO were younger (33.8 ± 13.4 vs. 37.0 ± 11.5 years, *p* = 0.02) but otherwise had similar demographic characteristics. Their rates of Roux-en-Y gastric bypass (39.4% SO vs. 44.4% NSO, *p* = 0.37) and sleeve gastrectomy (60.6% vs. 55.6%, *p* = 0.37) were similar. There were no differences between perioperative complications (6.4% SO vs. 5.8% NSO, *p* = 0.83), major postoperative complications (5.3% vs. 3.5%, *p* = 0.42), readmissions (5.3% vs. 3.3%, *p* = 0.36), or reoperations (3.2% vs. 2.7%, *p* = 0.78). There were no mortalities. Their total body weight loss was comparable at 12 months (28.1 ± 10.2% vs. 29.0 ± 7.7%, *p* = 0.58). **Conclusions**: Although a higher BMI may pose operative challenges, UAE patients with SO do not have worsened outcomes in bariatric surgery, demonstrating similarly low morbidity to patients with NSO, and similar rates of improvement in their BMI.

## 1. Introduction

The prevalence of obesity has been increasing worldwide, nearly tripling since 1975 [1]. In the United Arab Emirates (UAE), approximately 34% of the population has obesity, defined as a body mass index (BMI) ≥ 30 kg/m^2^; one of the highest rates of obesity in the Gulf Cooperation Council countries [2,3]. Additionally, there are high rates of cardiometabolic diseases including diabetes mellitus, hypertension, and metabolic syndrome in the UAE, which are associated with morbid obesity [4]. Most of the UAE population lives in urban areas and has a sedentary lifestyle, contributing to the doubling of obesity rates from 16 to 34% in the country since 2000 and class III obesity (BMI > 40 kg/m^2^) rising from 2 to 11% [5]. Similarly, the morbidity and mortality of cardiovascular disease have been rising at the same time, making the burden of obesity and cardiovascular risks a public health crisis that is drawing the attention of policymakers and several stakeholders [6].

Obesity and its associated medical problems have a negative impact on quality of life, patient activation, work productivity, and weight loss behaviors [7,8]. Lifestyle interventions including diet modification, exercise, and behavioral therapy can be beneficial in improving obesity and its related comorbidities [9]. However, many patients with obesity who have failed to lose weight with lifestyle and medical therapies need surgical intervention for a successful outcome. Bariatric surgery is an effective treatment for weight loss and the improvement of medical comorbidities in these individuals and is becoming increasingly more common [10,11]. Sleeve gastrectomy (SG) and Roux-en-Y gastric bypass (RYGB) are the most popular procedure choices, accounting for 53.6% and 30.1% of all bariatric procedures internationally [12,13].

Patients with severe obesity (SO) have a BMI ≥50 kg/m^2^ and often have a higher prevalence of cardiopulmonary disease and multiple comorbidities [14]. The anatomic and physiologic changes that occur in obesity can include altered wound healing due to the inherent anatomic features of adipose tissue, vascular insufficiencies, cellular and composition modifications, oxidative stress, alterations to immune mediators, and nutritional deficiencies [15]. These risk factors may predispose patients to an increased risk of postoperative complications such as respiratory failure, deep venous thrombosis, pulmonary embolism, anastomotic leaks, and increased bleeding, as well as increased morbidity and mortality, making them more challenging to treat than other patients [14]. However, the data that have been reported on individuals with SO are quite limited, especially within the Gulf population. Additionally, there is no agreement on the postoperative outcomes in patients with SO. In other populations, some studies have reported increased perioperative and postoperative complications in patients with SO compared to patients with non-severe obesity (NSO), defined as a BMI < 50 kg/m^2^, undergoing bariatric surgery [14,16]. Other reports have found only slightly higher morbidity and mortality rates than in patients with less severe obesity, but at an overall low incidence and with surgery still recommended [17]. In contrast, several studies report that bariatric surgery is just as safe in patients with SO and that these individuals can achieve successful weight loss outcomes and an improvement of their comorbidities [18,19,20,21,22]. These mixed data leave clinicians and surgeons without clear guidelines on whether or not to recommend a bariatric procedure to patients with SO. The UAE population is characterized by high rates of SO, especially in younger age groups. Therefore, there is a clear need to study this population to provide recommendations for current clinical practice in the UAE. This study aims to compare the perioperative and postoperative outcomes between patients with SO and NSO undergoing primary bariatric surgery at a tertiary referral medical center in the UAE.

## 2. Materials and Methods

### 2.1. Study Design

**Figure 1:**
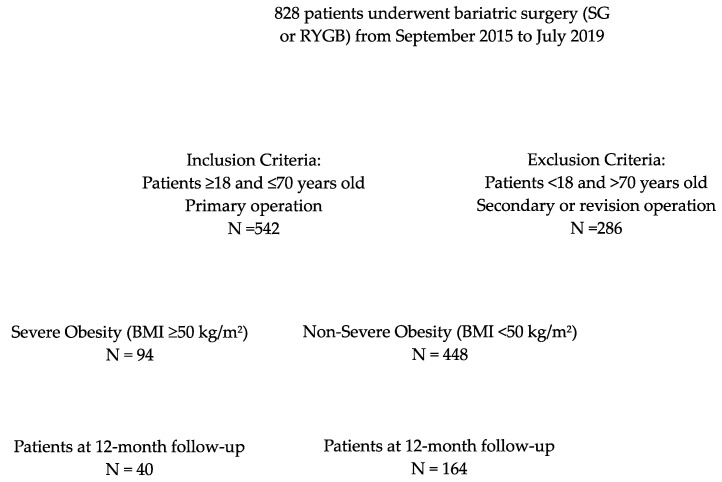
Study flow chart.

This retrospective study was conducted at a single academic medical institution located in the United Arab Emirates (UAE). All patients who underwent primary bariatric surgery at our institution between September 2015 and July 2019 were reviewed (Figure 1). This study was approved by our research ethical committee (REC) A 2017 029.

### 2.2. Aims

The primary aim was to compare the perioperative and postoperative outcomes between patients with SO and NSO undergoing primary bariatric surgery. The secondary aims were to compare the complication rates, conversion from laparoscopic to open surgery, and postoperative weight changes between the two groups.

### 2.3. Definitions

Severe obesity (SO): BMI ≥ 50 kg/m^2^.

*A BMI ≥ 50 kg/m^2^ may have been previously referred to as “super obesity”.

Non-severe obesity (NSO): BMI < 50 kg/m^2^.

Major complications: any complication that results in a prolonged hospital stay (>7 days), anticoagulant administration, reoperation, or reintervention [22].

Minor complications: any complication not considered major, such as transient nausea/vomiting, urinary tract infection, etc. [22].

Percentage of total body weight loss (%TBWL): calculated as (preoperative weight—follow up weight)/preoperative weight × 100.

### 2.4. Inclusion/Exclusion Criteria for Study Participation

All patients ≥18 and ≤70 years old undergoing primary SG or RYGB for the management of weight loss were included. Patients <18 or >70 years and patients undergoing revisional SG or revisional RYGB were excluded.

### 2.5. Preoperative Evaluation

Preoperative workup included an evaluation by our multidisciplinary team for patients undergoing bariatric surgery. Preoperative investigations comprised esophagogastroduodenoscopy (EGD), contrast-enhanced upper gastrointestinal series, and blood chemistry panels. Abdominal computerized tomography scans and/or abdominal ultrasounds were obtained at the discretion of the treating physicians.

### 2.6. Surgical Approach

Sleeve gastrectomy (SG) and Roux-en-Y gastric bypass (RYGB) were the two surgical approaches used. Selection of the surgical procedure was at the surgeon’s discretion and based on the specific patient’s needs, with a decision ultimately reached via shared decision making.

### 2.7. Institutional Surgical Techniques and Bariatric Procedures

SG: The patient was transported to the operating suite and identified by their full name, medical record identification, and birth date. A pre-operative team meeting, including the anesthesiology and surgical groups, was conducted. The patient was positioned horizontally on the surgical table. Their abdominal area was sanitized and covered following standard aseptic protocols. A surgical pause for final verification was observed. Entry into the peritoneal space was made using a 5 mm viewing trocar situated in the upper left abdominal quadrant. A pneumoperitoneum was created. Local anesthesia was applied bilaterally in the transversus abdominis plane. Trocar insertion followed a smooth U-shaped configuration.

A Nathanson liver retractor was applied under visual guidance. Dissection commenced with the removal of the phrenoesophageal adipose pad, revealing the angle of His. The greater curvature was freed by severing the gastrocolic ligament, proceeding distally and halting approximately 5 cm before the pyloric valve. The posterior short gastric vessels were severed to fully free the upper stomach section. With a 40 French Bougie in place, the gastric sleeve was fashioned using successive applications of mechanical staplers. Blood control was confirmed to be excellent. The excised tissue was extracted, and the entry site was sutured using a #0 Vicryl in a figure-eight configuration. An endoscope was inserted orally and guided visually to the duodenum to ensure the gastric sleeve was unobstructed and leak-free. Following the removal of the retractor, the pneumoperitoneum was released. The incisions were sutured using 4-0 Monocryl. The patient showed good tolerance to the procedure and was subsequently moved to the post-anesthesia care unit in stable condition.

RYGB: The procedure was initiated by transferring the patient to the operating area, where they were identified by their full name, medical record identifier, and date of birth. A pre-operative gathering involving the anesthesiology and surgery teams was conducted. The patient was laid horizontally on the operating platform, and their abdominal region was sterilized and covered as per the standard aseptic technique. A pause for a final procedural confirmation was observed. Access to the peritoneal cavity was gained via a 5 mm optical trocar placed in the left upper quadrant, establishing a pneumoperitoneum. Bilateral TAP blocks were administered. Trocars were situated following a gentle U-pattern and a Nathanson liver retractor was employed under visual guidance.

The surgical team proceeded to locate the left gastric pedicle and transect the descending branch just distal to it using a reinforced purple load. A series of endo-GIA stapler firings were employed to construct a diminutive gastric pouch. Subsequently, the ligament of Treitz was located, and, 100 cm distal to this landmark, the bowel was transected with a single application of the GIA stapler utilizing a tan load. Additional mesentery was dissected using an ultrasonic dissector. A Roux limb measuring 100 cm was prepared, and a side-to-side jejunojejunostomy was established with a single firing of the 60 mm GIA stapler, also with a tan load. The enterotomy was closed using a continuous 2-0 Vicryl suture.

Closure of the mesenteric defect was accomplished using 2-0 Ethibond. An omental division was executed. The team then created the gastrojejunostomy using a linear stapler and secured the Pseudo-Petersen’s defect with a continuous 2-0 Ethibond suture. Closure of all 12 mm trocar sites was achieved with a #0 Vicryl using the Carter–Thomason device. To assess the patency of the anastomosis, the bowel was clamped, and a front-viewing endoscope was introduced orally under direct vision, navigating through to the jejunum. The anastomosis was confirmed to be broadly unobstructed, with no signs of intraluminal hemorrhage or leakage. The retractor was removed, and the pneumoperitoneum was discharged. Incisions were sutured using 4-0 Monocryl. The patient demonstrated good procedural endurance and was subsequently moved to the post-anesthesia care unit in a stable condition.

### 2.8. Postoperative Care

Postoperatively, patients were admitted to the surgical ward under a standardized recovery protocol consisting of early ambulation, incentive spirometry, and drinking sips of water. Multimodal narcotic-sparing analgesia and venous thromboembolism prophylaxis were administered. A clear liquid diet was initiated after surgery and was advanced to full liquid on postoperative day two. After monitoring for perioperative complications, patients were discharged home when tolerating adequate oral intake. Patients were discharged on low-molecular-weight heparin for two weeks and multivitamins were prescribed. Patients were followed up through evaluation in our outpatient clinic with members of our multidisciplinary team from surgery, nutrition, and other medical specialties depending on the patient’s other comorbidities.

### 2.9. Data Collection and Surgical Outcomes

Data were collected retrospectively from electronic medical records and maintained in an institutional registry. Surgical outcomes included, but were not limited to, the length of stay, intensive care unit (ICU) admissions, emergency department (ED) visits, readmissions, reoperations, early complications, weight loss evolution, and mortality during the study period.

### 2.10. Statistical Analyses

Descriptive statistics were computed for all variables. Continuous variables were summarized using means and standard deviations or medians and interquartile ranges, and categorical variables were summarized using frequencies and percentages. Comparisons were made using parametric or non-parametric methods, where appropriate, and a significance level of *p* < 0.05 was used to determine statistical significance. Comparison tests included independent sample t-tests or Wilcoxon rank-sum (or Mann–Whitney U) tests when examining continuous variables, and Chi-square tests when examining dichotomous variables. All analyses were carried out using R (version 2.13, The R Foundation for Statistical Computing, Vienna, Austria).

## 3. Results

### 3.1. Demographic Characteristics

There were a total of 542 patients included in the study, with 94 patients (17.3%) in the SO cohort and 448 patients (82.7%) in the NSO cohort. The SO cohort was younger on average, with a mean age of 33.8 ± 13.4 years, whereas the NSO had a mean age of 37.0 ± 11.5 years (*p* = 0.02). The initial mean BMI for the SO cohort was 56.5 ± 6.2 kg/m^2^, while for the NSO cohort it was 41.8 ± 4.1 kg/m^2^ (*p* < 0.00001). The gender distribution was similar, as the SO cohort was 55.3% female and the NSO cohort was 62.9% female (*p* = 0.17). Importantly, both cohorts exhibited similar comorbidity profiles including comparable rates of hypertension, diabetes mellitus, and gastroesophageal reflux disease (GERD). The SO cohort did, however, have a higher rate of obstructive sleep apnea (47% SO vs. 31% NSO, *p* = 0.003) and the NSO cohort had a higher rate of hyperlipidemia (35% SO vs. 48% NSO, *p* = 0.02). Additional details of their comorbidities are described in Table 1. Regarding the surgical procedures, 39.4% of the SO cohort underwent RYGB, while 60.6% underwent SG. In the NSO cohort, 44.4% underwent RYGB and 55.6% underwent SG.

### 3.2. Operative Details

Notably, no statistically significant differences were observed in our intraoperative findings. The average operative time for RYGB was 159 ± 32 min for the SO cohort and 161 ± 58 min for the NSO cohort (*p* = 0.80). The average operative time for SG was 90 ± 20 min for the SO cohort and 92 ± 32 min for the NSO cohort (*p* = 0.70). Ninety-three (98.9%) cases were completed via a laparoscopic approach in the SO cohort and 442 (98.6%) cases in the NSO cohort (*p* = 0.83), with only one conversion in the NSO group due to bowel perforation. Intraoperative complications occurred in 6 (6.4%) cases in the SO cohort and 26 (5.8%) cases in the NSO cohort (*p* = 0.83). The estimated blood loss was <125 mL in all cases (Table 2).

### 3.3. Postoperative Complications

There were no statistically significant differences in the complication rates between cohorts. Early minor complications occurred in 5 (5.3%) patients in the SO cohort and 24 (5.3%) patients in the NSO cohort (*p* = 0.99), with the most common being transient nausea and vomiting. Early major complications occurred in 5 (5.3%) patients in the SO cohort and 16 (3.5%) patients in the NSO cohort (*p* = 0.42). More specifically, the rates of anastomotic leaks were extremely low (1.0% SO vs. 0.4% NSO, *p* = 0.47), as well as the rates of GI bleeding (1.0% SO vs. 1.5% NSO, *p* = 0.72). Detailed information describing the perioperative complications is included in Table 3.

### 3.4. Postoperative Outcomes

An analysis of the postoperative outcomes revealed no statistically significant differences between the two cohorts (Table 4). The mean length of their hospital stays did not differ significantly, with SO patients staying for an average of 3.1 ± 3.9 days compared to 2.7 ± 1.3 days for NSO patients (*p* = 0.06). Readmission rates within the early postoperative period were similar, at 5.3% for patients with SO and 3.3% for patients with NSO (*p* = 0.36). Furthermore, reoperation rates were low, at 3.2% for patients with SO and 2.7% for patients with NSO (*p* = 0.78). There were no mortalities in either cohort (Table 4).

### 3.5. Follow-up Outcomes

Patients in the SO cohort were found to be followed up with for a longer duration after their surgery, with an average follow-up time of 13.5 ± 11.0 months compared to 10.9 ± 9.2 months for the patients in the NSO cohort (*p* = 0.02). There were 40 (42.5%) patients reaching a 12-month follow-up in the SO cohort and 164 (36.6%) patients who did so in the NSO cohort (*p* = 0.28). Interestingly, both cohorts had similar changes in their weight loss at 12 months postop, for which the % TBWL was 28.1 ± 10.2% in patients with SO and 29.0 ± 7.7% in patients with NSO (*p* = 0.58) (Table 5).

## 4. Discussion

The burden of obesity is continuing to rise in the UAE and worldwide, in both number and severity. Patients with obesity often have a higher prevalence of cardiovascular disease, diabetes mellitus, and other comorbidities that may increase their risk of perioperative complications and poorer postoperative outcomes [4]. Some studies have previously shown that a greater BMI is an independent risk factor for morbidity and mortality in bariatric surgery [14,16,23]. It is not clear whether patients with SO (BMI ≥ 50 kg/m^2^) are at greater risk for complications than patients with NSO, and there are no established guidelines on whether to recommend a bariatric procedure for these patients.

In our series of patients in the UAE undergoing primary bariatric surgery, there were no notable differences in the perioperative and postoperative outcomes between patients with SO and NSO. Their perioperative major complications were comparable, with similarly low complication rates. Furthermore, the postoperative outcomes demonstrated similarly low numbers of readmission rates and reoperation rates in both cohorts. These results are important for this population because the UAE has high rates of SO, especially in younger age groups, and the demand for bariatric surgery is increasing [2,24]. The demand for bariatric surgery in the UAE is growing, with a projected compound annual growth rate (CAGR) of 5.9% over the next 5 years, and demand for it the Middle East, including the UAE and Africa, is predicted to surge to a CAGR of 8.74% from 2019 to 2028 [25,26]. However, data on patients with obesity in the Middle East are lacking and recommendations are not well described, especially compared to in other parts of the world.

The results of our study align with and build upon several studies from the USA and Europe that report that bariatric surgery is just as safe in patients with SO as in those with NSO and that these individuals can achieve successful weight loss and an improvement of their comorbidities [18,19,20,21]. A retrospective study in Europe, in 2011, by Dapri et al. reported the morbidity and mortality after bariatric surgery in 31 patients with BMIs > 50 kg/m^2^, with an average follow-up of 28 months [18]. They found acceptable morbidity, with a 13% early complication rate and a 10% late complication rate; effective weight loss, with a loss of excess weight of 54.8% ± 16%; and a resolution of 51.1% of comorbidities [18]. A retrospective study from 2011, in Europe, by Mukherjee et al. similarly demonstrated that surgery was safe and effective for weight loss in their 61 patients with BMIs ≥ 50 kg/m^2^, with a 9.8% complication rate, 39% excess body weight loss in 1 year, and a high percentage of comorbidity resolution [20]. Another retrospective study from 2015, in the USA, by Daigle et al. examined the perioperative and postoperative outcomes in 30 patients age >65 years with BMIs ≥ 50 kg/m^2^ and found no deaths, conversions, or intraoperative complications, a 10% early morbidity rate, and a change in total body weight of 24.4% ± 12.2% [19]. Further support comes from a prospective study from 2009, in Europe, by Torchia et al., who examined 823 patients with BMIs ≥ 50 kg/m^2^, 95 of which had BMIs > 60 kg/m^2^ [21]. They reported no mortality, intraoperative, or 30-day major complications and even showed that a BMI < 30 can be achieved with a multi-disciplinary follow-up [21]. Our study had low perioperative complications comparable to previous reports, at 5.3% in our SO cohort and 3.5% in our NSO cohort, and no mortalities within either group. Additionally, our BMI measurements at the 12-month postoperative follow-up demonstrated decreases in total body weight by almost 30% in both groups. Patients classified as having SO preoperatively improved their weight to a classification of NSO, on average, postoperatively (56.5 ± 6.2 to 40.8 ± 8.1 kg/m^2^), and those classified as having NSO preoperatively moved to a classification of overweight, on average, postoperatively (41.8 ± 4.1 to 29.4 ± 4.2 kg/m^2^). These outcomes support the effectiveness of bariatric surgery in helping patients with all types of obesity lose weight and, for the first time, in the Middle Eastern population.

This study is subject to some limitations, given its retrospective nature. There may be other confounding factors impacting the results that were not considered. Given the retrospective nature of this study, no sample size calculation was performed, as we included all possible patients during the review period who met inclusion criteria. Another limitation was the percentage of patients remaining at the 12-month follow-up and thereafter. The results of the surgery may be different with a longer follow-up, but this was unable to be captured in this study. Patient selection may be biased, given who can access care at a tertiary medical center in the UAE. Selection bias may limit the generalizability of our findings to the entire population or other countries in the region. This, however, also highlights a strength, in that this study provides insight into a population with obesity in the Middle East which has not been previously described. Finally, we recognize there is not a clear consensus on what is defined as “severe obesity” [27,28]. With recent terminology and definition changes, we chose to replace the previous term “super obesity” with “severe obesity”, defined as a BMI ≥ 50 kg/m^2^ in several other studies [14,16,18,19,20]. Our study compares those with BMIs ≥ 50 kg/m^2^ and BMIs < 50 kg/m^2^, which is important for readers to consider when comparing to other studies.

Overall, this study adds valuable information to the medical literature regarding surgical outcomes in SO. Its strengths include the large sample size, being the first study that we know of to describe bariatric surgery outcomes in patients with SO in the Middle East, the fact that our results are comparable to rigorous studies in other parts of the world, and that our patients are doing well postoperatively. The procedures included in this study, SG and RYGB, are also the two most common bariatric procedures, accounting for 53.6% and 30.1% of all bariatric procedures internationally [12,13]. This makes the findings more comparable to other studies and more applicable to clinical practice. These results are secondary to the strong bariatric protocols held by this institution in the UAE, which follows the guidelines provided by the International Federation for the Surgery of Obesity and Metabolic Disorders (IFSO) and the American Society for Metabolic and Bariatric Surgery (ASMBS). The guidelines provided by local authorities in the Middle East and the Department of Health of the UAE are extremely limited.

Therefore, the results from our study can be used to impact clinical care by contributing to the weight management recommendations for patients with SO in the region. Specifically, this information can help change guidelines regarding how and when patients are selected for bariatric surgery. Patients with SO who were thought to be high risk had no difference in their complications and outcomes from patients with NSO during and after surgery. The follow-up data also demonstrate similar changes in total body weight lost, supporting the effectiveness of surgery in aiding weight loss in these patients. Thus, patients with SO should not be excluded from bariatric surgical care. In conjunction with a multidisciplinary care team and shared decision making between patients and providers, bariatric surgery is helpful in improving the weight loss and overall health of patients with SO.

## 5. Conclusions

Although a higher BMI may theoretically pose operative challenges, patients in the UAE with SO do not have worsened outcomes in bariatric surgery, demonstrating similarly low morbidity to patients with NSO and similar rates of total body weight loss. Further studies and randomized control trials are warranted to confirm these findings, especially in the Middle East, and provide a strong foundation for informing clinical guidelines.

## Figures and Tables

**Table 1:** Baseline characteristics.

N = 542	SO (n = 94)	NSO (n = 448)	*p*-Value
Age, yr, mean ± SD	33.8 ± 13.4	37.0 ± 11.5	0.02
BMI, kg/m^2^, mean ± SD	56.5 ± 6.2	41.8 ± 4.1	<0.00001
Female, n (%)	52 (55.3)	282 (62.9)	0.17
ASA, median (Range)	2.7 (2–3)	2.6 (2–3)	0.10
Comorbidities, n (%)			
Obstructive sleep apnea	44 (46.8)	138 (30.8)	0.003
Hyperlipidemia	33 (35.1)	217 (48.4)	0.02
Hypertension	32 (34.0)	154 (34.4)	0.95
Diabetes mellitus	28 (29.8)	133 (29.7)	0.98
GERD	21 (22.3)	116 (25.9)	0.47
Chronic kidney disease	5 (5.3)	22 (4.9)	0.87
Coronary artery disease	2 (2.1)	20 (4.5)	0.30
End stage renal disease	1 (1.1)	3 (0.7)	0.68
COPD	0 (0)	8 (1.8)	0.19
Current smoker, n (%)	15 (15.9)	66 (14.7)	0.76
Type of bariatric surgery, n (%)			
RYGB	37 (39.4)	199 (44.4)	0.37
Sleeve gastrectomy	57 (60.6)	249 (55.6)	0.37

Abbreviations: SO = severe obesity; NSO = non-severe obesity; SD = standard deviation; BMI = body mass index; IQR = interquartile range; ASA = American Society of Anesthesiologists; GERD = gastro-esophageal reflux disease; COPD = Chronic obstructive pulmonary disease; RYGB = Roux-en-Y gastric bypass.

**Table 2:** Operative details.

N = 542	SO (n = 94)	NSO (n = 448)	*p*-Value
Laparoscopic surgical approach, n (%)	93 (98.9)	442 (98.6)	0.83
Conversion rate, n (%)	0 (0)	1 (0.2)	0.65
Complications, n (%)	6 (6.4)	26 (5.8)	0.83
Operative time, min (mean ± SD)			
RYGB	159 ± 32	161 ± 58	0.80
SG	90 ± 20	92 ± 32	0.70
Blood loss <125 mL (mean ± SD)	94 (100)	448 (100)	---

Abbreviations: SO = severe obesity; NSO = non-severe obesity; SD = standard deviation; RYGB = Roux-en-Y gastric bypass; SG = sleeve gastrectomy.

**Table 3:** Perioperative complications.

N = 542	SO (n = 94)	NSO (n = 448)	*p*-Value
Early minor complications, n (%)	5 (5.3)	24 (5.3)	0.99
Nausea, vomiting	4 (4.3)	15 (3.3)	0.67
Trocar/surgical site infection	1 (1.0)	3 (0.6)	0.68
Pneumonia	0 (0)	0 (0)	-
UTI	0 (0)	1 (0.2)	0.65
Dumping syndrome	0 (0)	2 (0.4)	0.52
Other	0 (0)	3 (0.6)	0.42
Early major complications, n (%)	5 (5.3)	16 (3.5)	0.42
VTE	0 (0)	0 (0)	-
Anastomotic leakage	1 (1.0)	2 (0.4)	0.47
GI bleeding	1 (1.0)	7 (1.5)	0.72
Postoperative transfusion	0 (0)	0 (0)	-
Small bowel obstruction	1 (1.0)	1 (0.2)	0.22
Bowel perforation	0 (0)	0 (0)	-
Surgical site infection	0 (0)	2 (0.4)	0.36
Myocardial infarction	0 (0)	0 (0)	-
Respiratory failure	1 (1.0)	1 (0.2)	0.22
Sepsis	0 (0)	2 (0.4)	0.52
Cerebrovascular accident	1 (1.0)	1 (0.2)	0.22
Endoscopy needed within 30 days	3 (3.1)	21 (4.6)	0.52
Placement of percutaneous drain	1 (1.0)	1 (0.2)	0.22

Abbreviations: SO = severe obesity; NSO = non-severe obesity; UTI = urinary tract infection; VTE = venous thromboembolism; GI = gastrointestinal.

**Table 4:** Postoperative outcomes.

N = 542	SO (n = 94)	NSO (n = 448)	*p*-Value
Length of stay, days (mean ± SD)	3.1 ± 3.9	2.7 ± 1.3	0.06
ED visits within 30 days, n (%)	18 (19.1)	112 (25)	0.27
Readmission within 30 days, n (%)	5 (5.3)	15 (3.3)	0.36
Reoperations within 30 days, n (%)	3 (3.2)	12 (2.7)	0.78
Mortality, n (%)	0 (0)	0 (0)	---

Abbreviations: SO = severe obesity; NSO = non-severe obesity; SD = standard deviation; ED = emergency department.

**Table 5:** Follow-up data.

N = 542	SO (n = 94)	NSO (n = 448)	*p*-Value
Duration of follow-up, months, mean ± SD	13.5 ± 11.0	10.9 ± 9.2	0.02
Patients at 12-month follow-up, N, %	40 (42.5)	164 (36.6)	0.02
BMI at 12 months postop, kg/m^2^, mean ± SD	40.8 ± 8.1	29.4 ± 4.2	<0.00001
% TBWL, mean ± SD	28.1 ± 10.2	29.0 ± 7.7	0.58

Abbreviations: SO = severe obesity; NSO = non-severe obesity; SD = standard deviation; BMI = body mass index; % TBML = percentage of total body weight loss.

## Data Availability

Data are contained within the article.
